# Transcriptome analysis of the sea cucumber (*Apostichopus japonicus*) with variation in individual growth

**DOI:** 10.1371/journal.pone.0181471

**Published:** 2017-07-17

**Authors:** Lei Gao, Chongbo He, Xiangbo Bao, Meilin Tian, Zhen Ma

**Affiliations:** 1 Key Laboratory of Marine Fishery Molecular Biology of Liaoning Province, Liaoning Ocean and Fisheries Science Research Institute, Dalian, China; 2 Dalian Fisheries Research Institute, Dalian, China; Dokuz Eylul Universitesi, TURKEY

## Abstract

The sea cucumber (*Apostichopus japonicus*) is an economically important aquaculture species in China. However, the serious individual growth variation often caused financial losses to farmers and the genetic mechanisms are poorly understood. In the present study, the extensively analysis at the transcriptome level for individual growth variation in sea cucumber was carried out. A total of 118946 unigenes were assembled from 255861 transcripts, with N50 of 1700. Of all unigenes, about 23% were identified with at least one significant match to known databases. In all four pair of comparison, 1840 genes were found to be expressed differently. Global hypometabolism was found to be occurred in the slow growing population, based on which the hypothesis was raised that growth retardation in individual growth variation of sea cucumber is one type of dormancy which is used to be against to adverse circumstances. Besides, the pathways such as ECM-receptor interaction and focal adhesion were enriched in the maintenance of cell and tissue structure and communication. Further, 76645 SSRs, 765242 SNPs and 146886 ins-dels were detected in the current study providing an extensive set of data for future studies of genetic mapping and selective breeding. In summary, these results will provides deep insight into the molecular basis of individual growth variation in marine invertebrates, and be valuable for understanding the physiological differences of growth process.

## Introduction

The sea cucumber (*Apostichopus japonicus*) belongs to family Holothuroidea with nutritional and supposed medicinal properties [1]. As a dominant aquaculture species in china, its aquaculture industry has grown rapidly over recent years. The annual production of sea cucumber had reached 200,000 tons in China by 2014, with the culture area exceeding one million acres and a production value over 3,000,000,000 dollars [2,3]. Nevertheless, serious individual growth variation is often found in both nature and culture populations of sea cucumber. The coefficient of variation (CV) of growth is 7–10% in farm animals and 20–35% in most fish species [4]. However, the individual growth variation of sea cucumber is often over 50%, and the growth retardation is very common in aquaculture practices forming the slow growing population [5]. Individual growth variation may affect the cultural production and cause financial losses to farmers.

Individual growth variation may result from genetic and environmental differentiation. Dong et al. reported that individual growth variation of sea cucumber increased with the increase of stocking densities, whereas no significant differences in variation were found when the density was over 30 inds [5]. Pei et al. gave a similar result, which showed that physical contact caused by stocking density could affect the individual growth variation of sea cucumber [6]. However, the genetic factor was thought to be more effective than stocking density [6]. Liang et al. reared sea cucumber individually to eliminate possible effects of environmental differentiation, such as social interaction, stocking density, etc. [7]. The result showed that the coefficient of variation in body weight increased from 12.04% to 40.51% during the 100-day experiment, which implies that genetic differences among sea cucumber individuals are the key factors accounting for the individual growth variation of sea cucumber [7]. Although advances have been achieved in the environmental study of sea cucumber individual growth variation, there is very limited information in terms of mechanisms and molecules involved in the processes.

The advent of next-generation RNA sequencing (e.g., Illumina mRNA-Seq) technology have greatly improved the understanding of functional complexity of transcriptome, especially de novo transcriptome sequencing for non-model organisms [8–11]. Using RNA-Seq technology, transcriptomic information have been obtained to identify the growth-related genes in aquatic species such as Nile tilapia (*Oreochromis niloticus*) [12], Abalone (*Haliotis midae*) [13] and the swimming crab (*Portunus trituberculatus*) [14]. In sea cucumber, these technologies have been used to understand transcriptomic information, focusing on different biological issues such as skin ulceration syndrome, regeneration and aestivation [15–17]. However, very little is known regarding the transcriptomic information involved in the sea cucumber individual growth variation.

In the present study, the body wall and intestinal transcriptome of sea cucumber with different growth rate was analyzed by Illumina sequencing and bioinformatics analysis. This is the first report on characterizing the sea cucumber transcriptome with individual growth variation, aiming to identify candidate growth-related genes and investigate potential growth molecules. The results provide valuable leads for further investigation of growth mechanism in sea cucumber, and improve the current understanding of individual growth variation.

## Materials and methods

### Ethics statement

Sea cucumber handling was conducted in accordance with the guidelines and regulations established by Liaoning Ocean and Fisheries Science Research Institute and the local government. The field studies did not involve endangered or protected species.

### Sample preparation

The sea cucumber individuals were obtained from six tanks of Breeding Center of Liaoning Ocean and Fisheries Science Research Institute. The individuals in each tank were from the same population, and there are three different populations for each of one-age and two-age individuals. Based on the significant individual growth variation, in this study, the top 50% of individuals sorted by weight were defined as normal growing, and the last 20% were defined as slow growing individuals. All of the individuals in six tanks were cultured under the same conditions for one or two years. Three groups of sea cucumber were used in this study, including AJ_2S (two-age slow growing individuals; length: 3±0.5 cm, weight: 10±3 g), AJ_2L (two-age normal growing individuals; length: 15±3 cm, weight: 100±15 g) and AJ_1S (one-age normal growing individuals; length: 5±1 cm, weight: 15±3 g). In each group, 15 individuals were sacrificed (five individuals from each tank) and two issues (body wall (represented by B) and intestine (represented by I)) were immediately removed and frozen in liquid nitrogen and kept at -80°C until use. All of the operations in this study were carried out in Liaoning Ocean and Fisheries Science Research Institute, and the permission had been given to conduct this study.

### RNA isolation, cDNA library construction and Illumina deep sequencing

Total RNA was isolated with Trizol Reagent (Invitrogen, USA). RNA degradation and contamination was monitored with 1% agarose gels. RNA purity was assessed using the NanoPhotometer spectrophotometer (IMPLEN, CA, USA). RNA concentration was tested using Qubit RNA Assay Kit (Invitrogen, USA). RNA integrity was checked in the Agilent Bioanalyzer 2100 system (Agilent Technologies, CA, USA). A total amount of 3 μg RNA per sample was used as input material for the RNA sample preparations. Equal amount of RNA from five individuals in each group were pooled as one sample, and three libraries were made for each group [18]. The sequencing libraries were prepared using NEBNext Ultra^™^ RNA Library Prep Kit for Illumina (NEB, USA) following manufacturer’s recommendations. The index codes were added to attribute sequence to each sample. The library fragments were purified using AMPure XP system (Beckman Coulter, Beverly, USA) to select cDNA fragments of preferentially 150~200 bp in length. PCR was performed using Phusion High-Fidelity DNA polymerase, Universal PCR primers and Index Primer. The products were purified in AMPure XP system and library quality was checked with Agilent Bioanalyzer 2100 system. The index-coded samples were clustered on a cBot Cluster Generation System using TruSeq PE Cluster Kit v3-cBot-HS (Illumina) according to the manufacturer’s instructions. Transcriptome sequencing of the library preparations was carried out on an Illumina HiSeq 2000 platform by Novogene (Beijing, China) to obtain paired-end reads (S1 Fig).

### Transcriptome assembly

Clean data were obtained by removing reads containing adapter, ploy-N and low quality reads from raw data through in-house perl scripts, and assembled into contigs using Trinity [19,20]. Q20, Q30 and GC-content of data were calculated for downstream analyses. The transcriptome sequence data have been deposited into the NCBI Sequence Read Archive (SRA) under the accession number of SRR5282240, SRR5282239, SRR5282238, SRR5282237, SRR5282236 and SRR5282235. The assembled sequences have been deposited in the NCBI Transcriptome Shotgun Assembly (TSA) database. This Transcriptome Shotgun Assembly project has been deposited at DDBJ/ENA/GenBank under the accession GFKU00000000. The version described in this paper is the first version, GFKU01000000 (S2 and S3 Figs).

### Bioinformatics analysis

For functional annotation, unigenes were subjected to the following databases: NCBI non-redundant protein sequences (Nr), NCBI non-redundant nucleotide sequences (Nt), SwissProt (amanually annotated and reviewed protein sequence database), Gene Ontology (GO), Kyoto Encyclopedia of Genes and Genomes (KEGG), clusters of orthologous groups of proteins (KOG/COG), Ortholog Database (KO) and protein family (PFam), using a cut-off E-value of 10^−5^. If confliction occurred among the results of different databases, a priority order of alignments from Nr, Nt, KEGG, Swiss-Prot, GO and COG databases was followed. NCBI blast 2.2.28+ was used for Nr, Nt, SwissProt and KOG annotation. HMMER 3.0 package hmmscan was used for PFAM annotation. GO annotation was carried by Blast2GO version 2.5 [21]. KAAS (http://www.genome.jp/tools/kaas/) and KEGG Automatic Annotation Server (http://www.genome.jp/kegg/) were used for KEGG annotation. Simple sequence repeats were identified using MISA (http://pgrc.ipk-gatersleben.de/misa/, version: 1.0). Primer3 (http://primer3.sourceforge.net/releases.php, version: 4.0.0) was used for primer design of SSRs. Gene expression levels based on read counts obtained by RSEM (version v1.2.15) were normalized using the FPKM (Fragments Per Kilo bases per Million fragments) transformation. Differential expression genes (DEGs) were screened using R package DEGseq based on the read count for each gene. Growth variation related genes were manually identified according to annotation in different signaling pathways. Trinity software was also used to predict protein coding sequences (CDSs).

### Real-time PCR assays

Eight annotated DEGs related to growth variation (Toll-like receptor 2, Follistatin, profiling, Ankyrin-1, Melanotransferrin, Stromelysin-2, Pacifastin proteinase inhibitor and Lactadherin) were selected randomly to validate the RNA-Seq results between AJ_2SB and AJ_2LB using Real-time PCR. Prepared total RNA used to synthesize the cDNA were the same as those used in Illumina sequencing. Primers were designed according to RNA-Seq data with Primer Premier 5. The Real-time PCR analysis was performed in Mx3005p^™^ Real-time thermal cycler with the PrimeScript^™^ RT Reagent Kit (TaKaRa). Beta-actin was used as an internal control to normalize the expression level, the transcription stabilities of which were validated as reference genes in previous study of AJ_2SB and AJ_2LB. The reaction mixture was comprised of 10 μL 2X SYBR^®^ Premix Ex Taq^™^, 0.8 μM of each primer, 1 μL cDNA template, and then filled to a final volume of 20 μL with distilled water. The amplification conditions were as follows: denaturation and enzyme activation at 95°C for 30 s, followed by 40 cycles of 94°C for 5 s, 55°C for 20 s, and 72°C for 20 s. Each sample was run in triplicate along with the internal control gene. A dissolution curve analysis of the amplification products was performed to confirm the specificity of the Real-time PCR products. The CT method (2-ΔΔCt) was used to calculate relative changes in My-Clp1 mRNA expression [22]. Statistical analysis was performed by one-way ANOVA of SPSS 13.0 software. *P*<0.05 and *P*<0.01 were set as highly significant and significantly different, respectively.

### SSR validation and polymorphism evaluation

The software MISA (http://pgrc.ipk-gatersleben.de/misa/misa.html) was used to identify microsatellite sequences from unigenes sequences. The software Primer 3 was used to design primer of each SSR. Compared with dinucleotide, of which the minimum number of repetitions was set at six, trinucleotide, tetranucleotide, pentanucleotide, and hexanucleotide were set at five. The maximum difference between two SSRs was set at 100 bp.

### SNP validation

Picard-tools v1.41 and samtools v0.1.18 were used to sort, remove duplicated reads and merge the bam alignment results of each sample. GATK2 software was used to perform SNP calling. Raw vcf files were filtered with GATK standard filter method and other parameters (clusterWindowSize: 10; MQ0 > = 4 and (MQ0/(1.0*DP)) > 0.1; QUAL < 10; QUAL < 30.0 or QD < 5.0 or HRun > 5), and only SNPs with distance > 5 were retained.

## Results

### Sequence analysis and assembly

This sequencing run produced about 3.2×10^8^~4.7×10^8^ paired-end raw reads in each library with an average Q20 equal to 97.8%. After filtering out low quality reads (adaptor sequences, ambiguous nucleotides and low-quality sequences), approximate 96% high-quality reads were obtained as clean reads. From the trimmed and size-selected reads, 255861 transcripts were assembled using the Trinity. The length of transcripts ranged from 201 to 44955 with N50 of 2082. These transcripts are subsequently assembled into 118946 unigenes, with N50 of 1700, and average length of 800.

### Functional annotation

**Fig 1:**
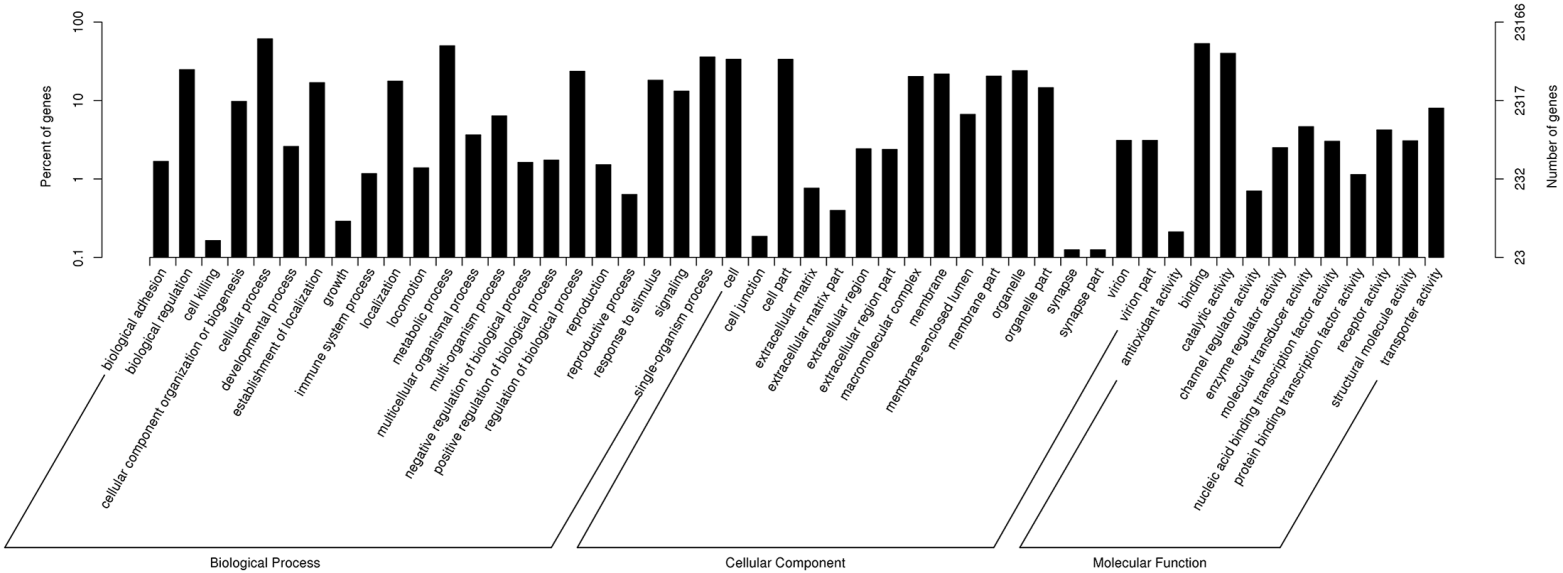
GO (Gene Ontology) categorization (Biological process, Cellular component and Molecular function) of unigenes in sea cucumber. Each annotated sequence is assigned at least one GO term. The x-axis represents GO subcategories. The left y-axis indicates the percentage of genes. The right y-axis indicates the number of DEGs.

**Fig 2:**
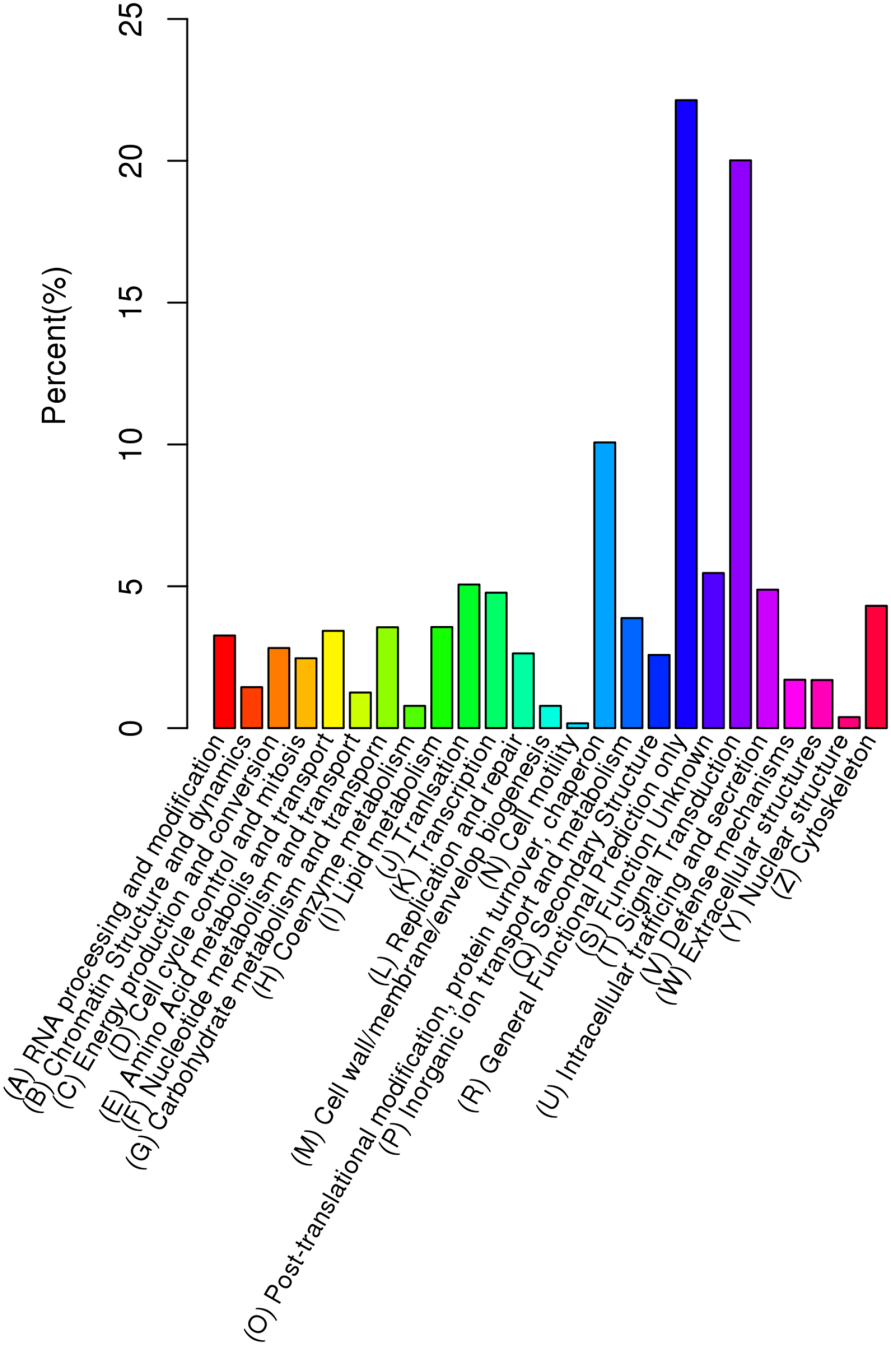
KOG classification of putative proteins.

All unigenes were subjected to annotation analysis by matching sequences against 7 databases. In detail, 19,240 unigenes (16.17% of the total), 3,256 unigenes (2.73% of the total), 5,155 unigenes (4.33% of the total), 15,336 unigenes (12.89% of the total), 22,625 unigenes (19.02% of the total), 23,166 unigenes (19.47% of the total) and 11,081 unigenes (9.31% of the total) can be matched in Nr, Nt, KO, SwissProt, PFAM, GO, and KOG databases, respectively (Figs 1 and 2). Of 118,946 unigene sequences, 28,243 (23.74% of all unigenes) had at least one significant match. A large number of unigenes (76.26% of all unigenes) were showed to have no significant match to known sequences.

### Identification of DEGs

**Fig 3:**
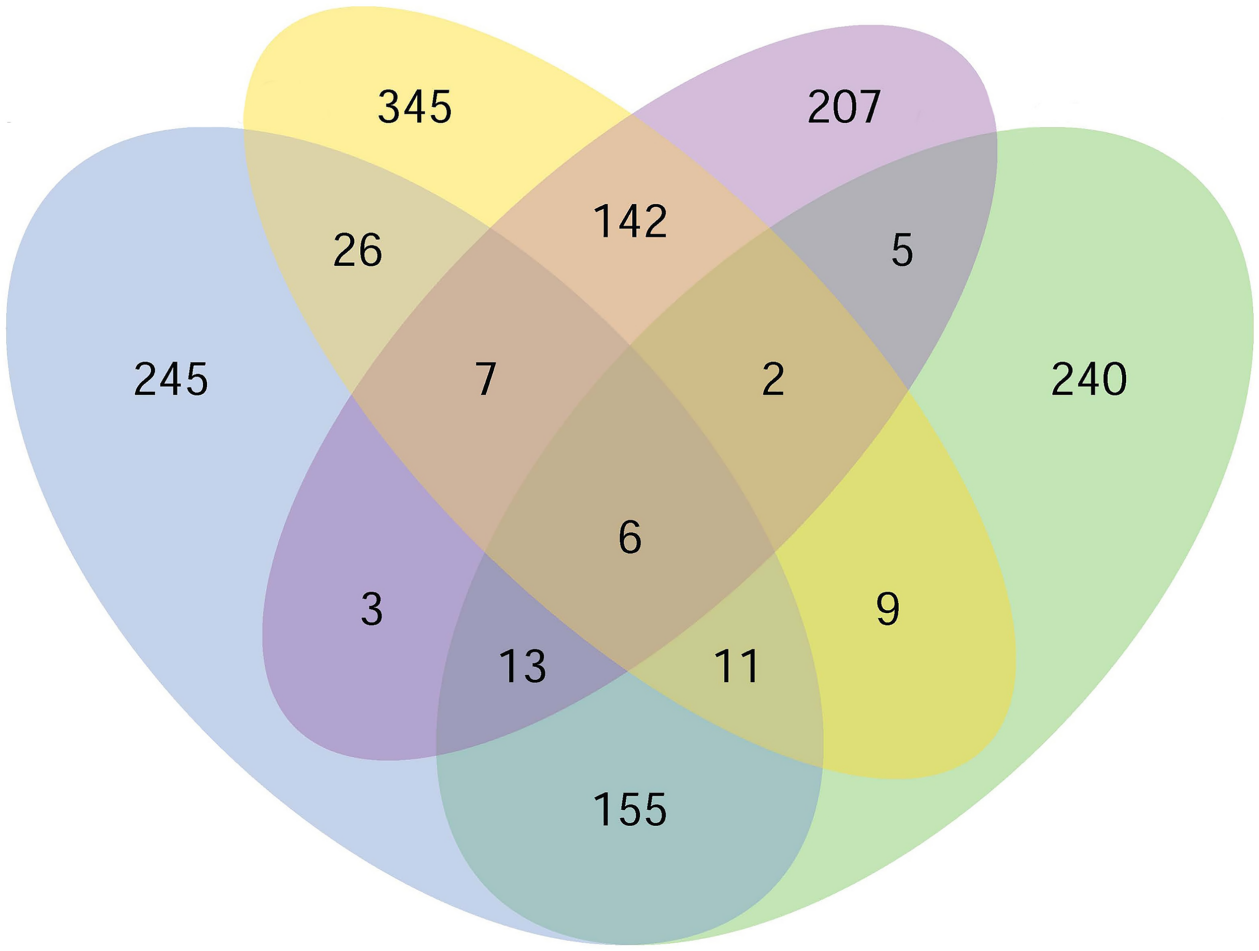
Venn diagram of differential gene expression in body wall and intestine of sea cucumber with different growth rate. The different colors represent four different subsets: blue indicates AJ_2SB vs. AJ_1SB; yellow indicates AJ_2SI vs. AJ_1SI; purple indicates AJ_2SI vs. AJ_2LI; green indicates AJ_2SB vs. AJ_2LB.

The significant differences in the expression of different unigenes were determined by quantifying the abundance of reads belonging to the same gene from the transcriptome between AJ_2S and AJ_2L, and between AJ_2S and AJ_1S (Fig 3). DEGs were screened following the criteria of |log2FoldChange|>1 and qvalue<0.005. In total, 441, 385, 466 and 548 DEGs were identified from the comparisons of AJ_2SB vs. AJ_2LB, AJ_2SI vs. AJ_2LI, AJ_2SB vs. AJ_1SB and AJ_2SI vs. AJ_1SI, respectively. For AJ_2SB vs. AJ_2LB, we detected 257 up-regulated DEGs and 184 down-regulated DEGs, whereas the AJ_2SI vs. AJ_2LI comparison produced 167 up-regulated DEGs and 218 down-regulated DEGs, and AJ_2SB vs. AJ_1SB comparison yielded 189 up-regulated DEGs and 277 down-regulated DEGs, and AJ_2SI vs. AJ_1SI comparison produced 150 up-regulated DEGs and 398 down-regulated DEGs. The key DEGs associated with cellular processes were showed in Table 1.

**Table 1:** Key DEGs associated with cellular processes.

Group and gene	Gene ID	AJ_2S (FPKM)	AJ_2L (FPKM)	AJ_1S (FPKM)
**Focal adhesion**				
Proto-oncogene tyrosine-protein kinase Fyn	comp314575_c0	19.07	33.44	44.52
**Tight junction**				
Serine/threonine protein kinase	comp300900_c0	112.91	21.6	70.91
Myosin heavy chain	comp304319_c0	2260.87	2789.79	4179.99
**Apoptosis**				
Caspase 6	comp296962_c0	30.24	6.08	82.37
**Cell cycle**				
Cyclin B	comp313379_c0	40.53	25.73	15.02
Polo-like kinase 1	comp313898_c0	24.69	16.4	10.97
Branchiostoma floridae hypothetical protein	comp318031_c0	23.24	13.54	10.47
**Regulation of actin cytoskeleton**				
Profilin	comp293611_c2	845.9	1089.64	1036.07
Myosin regulatory light chain 2	comp311234_c0	4482.37	4894.45	7715.16

### Gene ontology analysis

To gain insights into the functional categories of transcripts, the unigenes with matches in public databases were annotated using Gene Ontology (GO) annotation, which is an international standardized gene functional classification system providing structured, controlled vocabularies and classifications (Fig 1, S1 Table).

Among the 118,946 unigenes, 23,166 were assigned at least one GO term, with a total of 139,485 GO assignments. Three major functional categories were obtained in assignments: cellular component (GO ID: 0005575; 43,423, 31.13%), biological process (GO ID: 0008150; 68,084, 48.81%) and molecular function (GO ID: 0003674; 27,978, 20.06%).

In the category of cellular component, dominant subcategories were cell (GO ID: 0005623; 7,776, 17.91%), cell part (GO ID: 0044464; 7,767, 17.89%), and organelle (GO ID: 0043226; 5,559, 12.80%). In biological process, genes involved in cellular process (GO ID: 0009987; 14,179, 20.83%), metabolic process (GO ID: 0008152; 11,558, 16.98%) and single-organism process (GO ID: 0044699; 8,322, 12.22%) were the most abundant. For molecular functions, the most represented GO terms were binding (GO ID: 0005488; 12,323, 44.05%) and catalytic activity (GO ID: 0003824; 9,258, 33.09%). The significantly enriched GO terms in DEGs was showed in Table 2.

**Table 2:** Significantly enriched GO terms in DEGs.

Gene ontology term	GO No.	Term type	DEGs numbers	Corrected P-Value
**Down-regulated in AJ_2SB vs. AJ_2LB**				
Carbohydrate binding	GO:0030246	Molecular function	10	0.0054205
**Down-regulated in AJ_2SI vs. AJ_2LI**				
Serine-type endopeptidase inhibitor activity	GO:0004867	Molecular function	7	0.0022839
**Up-regulated in AJ_2SI vs. AJ_1SI**				
Oxidoreductase activity	GO:0016491	Molecular function	24	0.01311
Cofactor binding	GO:0048037	Molecular function	12	0.032281
**Down-regulated in AJ_2SI vs. AJ_1SI**				
Peptidase inhibitor activity	GO:0030414	Molecular function	15	0.00020264
Peptidase regulator activity	GO:0061134	Molecular function	15	0.00020264
Endopeptidase inhibitor activity	GO:0004866	Molecular function	13	0.00026649
Endopeptidase regulator activity	GO:0061135	Molecular function	13	0.00026649
Enzyme inhibitor activity	GO:0004857	Molecular function	17	0.00054613
Serine-type endopeptidase inhibitor activity	GO:0004867	Molecular function	8	0.0051751

The terms with corrected P-value<0.05 were considered as the enriched ones. The categories not listing are with no terms significantly enriched.

### Pathway enrichment analysis

**Fig 4:**
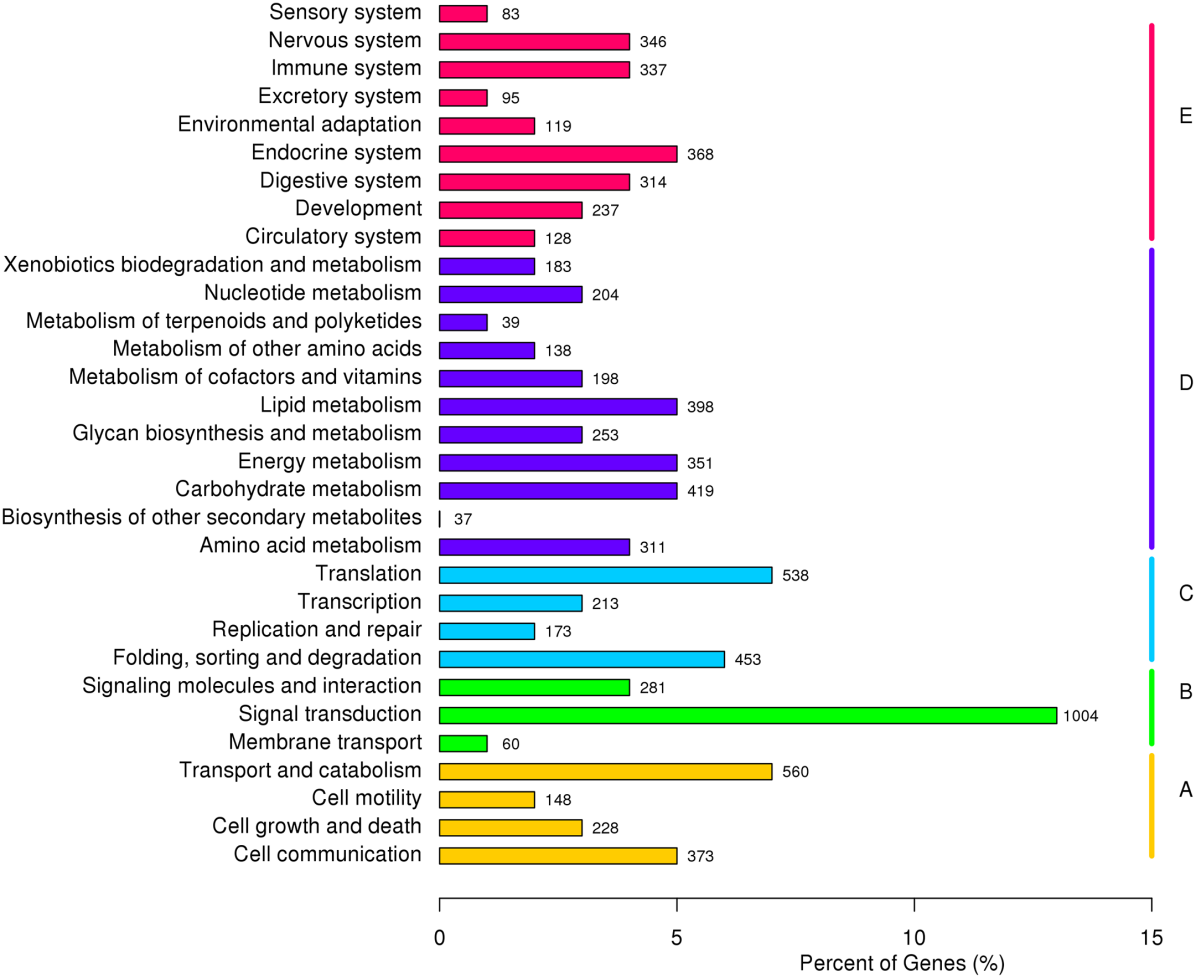
KEGG annotation of putative proteins. A, Cellular Processes; B, Enviromental Information Processing; C, Genetic Information Processing; D, Metabolism; E, Organismal Systems.

KEGG is a pathway-based categorization recording networks of molecular interactions [23]. To further explore the biological pathways and analyze the interplay among DEGs, the annotated CDS sequences of all unigenes were mapped to the referential canonical pathways in the KEGG database (Fig 4). A total of 11,322 unigenes were associated with 5 pathway groups, including cellular processes (1,617, 14.28%), environmental information processing (1,931, 17.06%), genetic information processing (1,515, 13.38%), metabolism (3,408, 30.10%) and organismal systems (2,851, 25.18%) (S2 Table).

In the 2^nd^ hierarchy pathway, 1,004 members assigned to “signal transduction”, followed by “transport and catabolism” (560 members), “translation” (538 members), “folding, sorting and degradation” (453 members), “carbohydrate metabolism” (419 members), “lipid metabolism” (398 members), “cell communication” (373 members), “endocrine system” (368 members), “energy metabolism” (351 members), “Nervous system” (346 members) and others. The enriched pathways in DEGs was showed in Table 3.

**Table 3:** Enriched pathways in DEGs.

Pathway term	Pathway ID	P-value	Corrected P-Value	SN/BN
**Down-regulated DEGs in AJ_2SB vs. AJ_2LB**				
ECM-receptor interaction	ko04512	7.16E-08	1.15E-05	11/97
Focal adhesion	ko04510	5.02E-06	4.04E-04	13/209
PI3K-Akt signaling pathway	ko04151	5.76E-05	3.09E-03	14/301
**Up-regulated DEGs in AJ_2SI vs. AJ_2LI**				
Ribosome biogenesis in eukaryotes	ko03008	5.76E-08	9.28E-06	9/64
Fatty acid degradation	ko00071	1.78E-04	1.44E-02	6/70
**Down-regulated DEGs in AJ_2SI vs. AJ_2LI**				
Glycerophospholipid metabolism	ko00564	1.55E-07	2.50E-05	9/78
alpha-Linolenic acid metabolism	ko00592	5.71E-07	3.80E-05	6/29
Linoleic acid metabolism	ko00591	7.08E-07	3.80E-05	6/30
Ether lipid metabolism	ko00565	1.36E-05	5.48E-04	5/29
Arachidonic acid metabolism	ko00590	6.04E-05	1.95E-03	6/63
Fatty acid metabolism	ko01212	1.68E-03	4.50E-02	5/79
**Down-regulated DEGs in AJ_2SB vs. AJ_1SB**				
ECM-receptor interaction	ko04512	7.16E-05	9.77E-03	9/97
Carbon fixation in photosynthetic organisms	ko00710	1.21E-04	9.77E-03	7/61
Glycolysis / Gluconeogenesis	ko00010	3.81E-04	1.94E-02	8/96
Focal adhesion	ko04510	4.82E-04	1.94E-02	12/209
Biosynthesis of amino acids	ko01230	1.13E-03	3.63E-02	8/113
Carbon metabolism	ko01200	1.80E-03	4.84E-02	9/150
**Up-regulated DEGs in AJ_2SI vs. AJ_1SI**				
Glycerolipid metabolism	ko00561	6.25E-05	7.73E-03	7/49
Fatty acid degradation	ko00071	9.60E-05	7.73E-03	8/70
One carbon pool by folate	ko00670	4.43E-04	2.38E-02	4/18
Glutathione metabolism	ko00480	8.44E-04	3.40E-02	7/74
**Down-regulated DEGs in AJ_2SI vs. AJ_1SI**				
Glycerophospholipid metabolism	ko00564	1.55E-07	2.50E-05	9/78
alpha-Linolenic acid metabolism	ko00592	5.71E-07	3.80E-05	6/29
Linoleic acid metabolism	ko00591	7.08E-07	3.80E-05	6/30
Ether lipid metabolism	ko00565	1.36E-05	5.48E-04	5/29
Arachidonic acid metabolism	ko00590	6.04E-05	1.95E-03	6/63

The pathways with corrected P-value<0.05 were considered as the enriched ones. The categories not listing are with no pathways significantly enriched. SN/BN: Sample number/Background number.

### Expression validation

**Fig 5:**
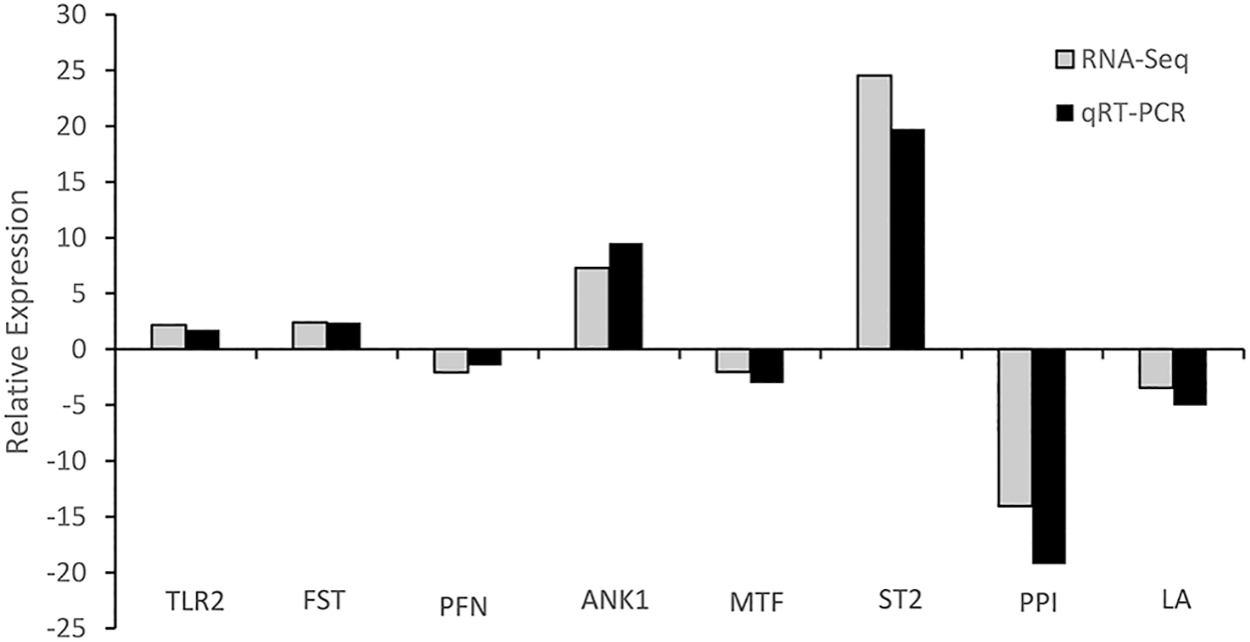
Comparison of relative fold changes between RNA-seq and qRT-PCR results between AJ_2SB and AJ_2LB. Positive values represent the up-regulated expression level in AJ_2SB; negative values represent the down-regulated expression level in AJ_2SB. TLR2: toll-like receptor 2; FST: follistatin; PFN: profilin; ANK1: ankyrin-1; MTF: melanotransferrin; ST2: stromelysin-2; PPI: pacifastin proteinase inhibitor; LA: lactadherin.

To validate the RNA-Seq results, eight unigenes were selected randomly to be analyzed using Real-time PCR. Among these selected genes, four were up-regulated genes (toll-like receptor 2, follistatin, ankyrin-1 and stromelysin-2) and another four were down-regulated genes (profiling, melanotransferrin, acifastin proteinase inhibitor and lactadherin) (Fig 5). The Real-time PCR results comfirmed the data obtained from Illumina sequencing analysis, which showed similar trends in up- or down-regulation of selected genes.

### Transcriptome-derived molecular markers development

**Fig 6:**
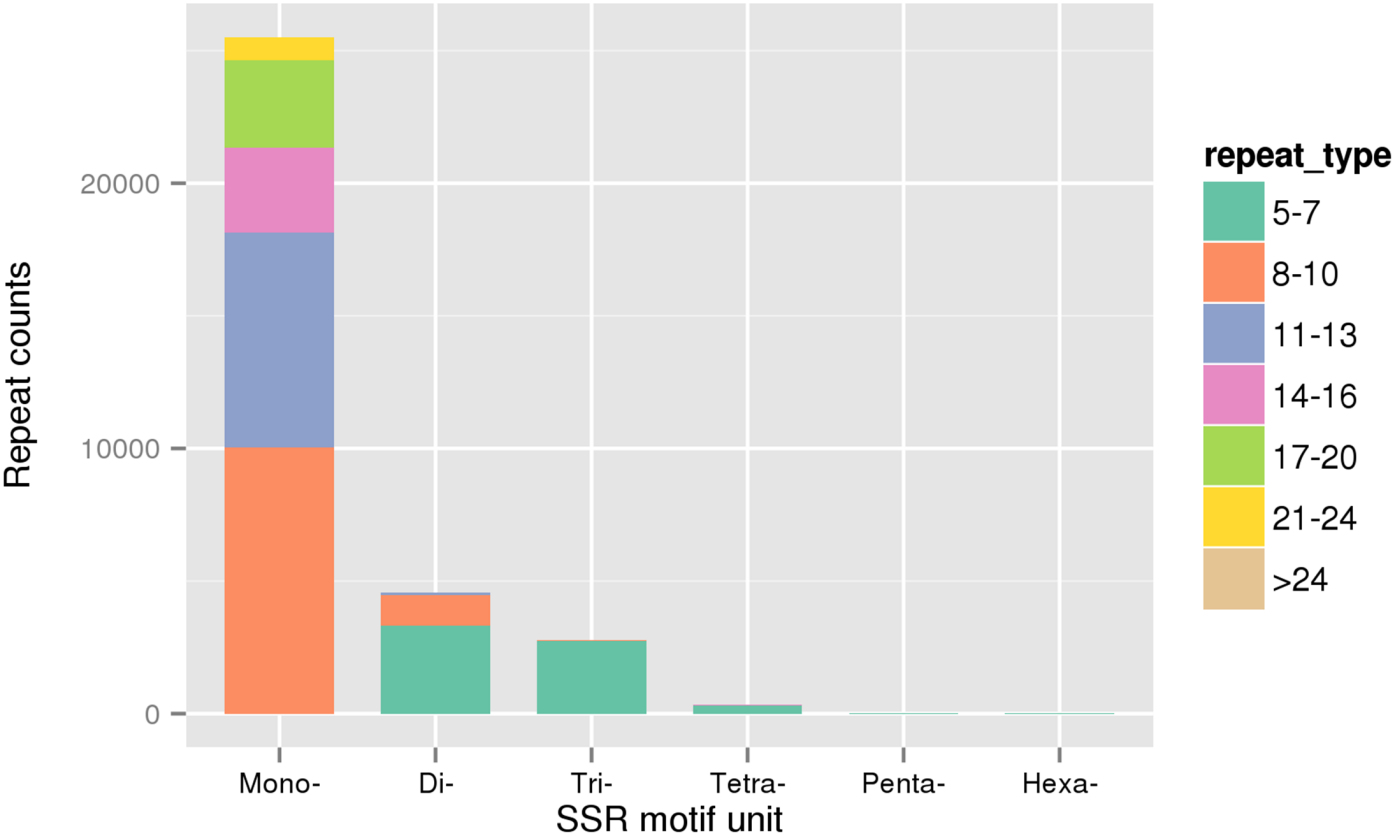
Distribution of SSR motifs. Different colors represent different repeat types.

In total, 76,645 SSRs located in 23,690 sequences were identified in the transcriptome dataset (Fig 6). The most common repeat motif was dinucleotide repeat (45,503) that accounted for 59.37% of the total SSRs, followed by trinucleotides (27,784, 36.25%), tetranucleotides (3,255, 4.25%), pentanucleotides (96, 0.13%) and hexanucleotides (7, 0.01%). Based on the distribution of SSR motifs, TG (391), ATT (153) and TACA (13) motifs were found to be the most common in di-nucleotides, tri- and tetra-nucleotides, respectively. SNPs were obtained from alignments of multiple sequences used for assembly. By excluding the sequences with distance lower than 5, a total of 765,242 SNPs and 146,886 ins-dels were identified. In SNPs, 26.86~31.11% were located on the coding region, of which about 0.03% were nonsynonymous. The identification of molecular markers provides an extensive set of data for future studies of genetic mapping and selective breeding.

## Discussion

Understanding the molecular mechanism in individual growth variation is very useful, not only for the studies on growth and development of sea cucumber, but also for selective breeding of fast growing strains. However, studies have been limited by the lack of sufficient knowledge in genomic resources. RNA-Seq is developed for transcriptome profiling using deep-sequencing technologies. With no strict requirement for genomic information, RNA-Seq is likely to become the main platform for global studies on the analysis of gene expression, differences of splicing activity, and exploration of SNPs in non-model organisms [24,25]. In this study, we investigated the transcriptome profile of sea cucumber with individual growth variation using the Illumina platform.

The large-scale gene expression was investigated among the sea cucumber individuals with growth variation for the first time. With any read been sequenced, the results indicated the sequencing saturation with sufficient depth of coverage, which could meet the needs of further analysis. Totally, 118,946 unigenes were assembled. However, about 76.26% of unigenes failed to match to the known protein database representing potentially novel genes, blasting limitation or sequencing errors. Some of the unmapped reads were expressed differentially. Further investigation, such as obtaining the complete genomic sequence, will help to improve this understanding. To further confirm gene expression profiles, the expression levels of 8 selected genes were tested using Real-time PCR. The results revealed that the expression trends analyzed by Real-time PCR were similar to those obtained by Illumina sequencing, whereas the amplitude of expression differences of several genes had discrepancies. This may be attributed to the sensitivity differences of the two methods. RNA-Seq has been reported to be more sensitive for assessing gene expression, especially for low-abundance transcripts [26,27].

High-throughput sequencing effort revealed that a number of molecules were highly enriched in metabolism, cellular processes and signaling pathways. Given this understanding, gene expression profiling and pathway analysis has contributed greatly to the investigation of regulatory mechanisms and understanding of global molecular variation. In this study, a number of DEGs were found to be differentially expressed among individuals with growth variation. Some genes encoding immunity defense (Interleukin, Toll-like receptor, cubilin, Adenosylhomocysteinase B, FGFR1 and Ponericin L) were up-regulated in AJ_2S vs. AJ_2L, which indicated that they may play an important role in intracellular defense against stress. Up-regulation of the genes associated with negative regulation of transcription & translation (EIF4, Hyalin, GPAT1, 3β-HSD) and down-regulation of ribosome biogenesis related genes (RPL32e, rpl21, rpl36 and RpP1) suggested that protein synthesis process were suppressed in AJ_2S, which was consistent with the previous studies [5]. Some life sustaining associated genes, like biosynthetic process (VTG, ATP synthase protein 8, PfPMT, MDH, transcription factor AP-1-like, hydroxyacid oxidase 1-like, Dehydrogenase, DMGDH), cell cycle (cyclin D, fibrillin-2-like, C-type lectin, Antistasin), metabolic process (Aqualysin-1, acetylcholinesterase, IαTI, BHMT1L, PTPs, complement component C3, ATP-citrate synthase, tyrosine-protein kinase Srms-like, ESPL, alkaline phosphatase) and cell proliferation (Fibropellin-1, autocrine proliferation repressor protein A-like, Inhibin β), showed reduced mRNA levels in AJ_2S, which implied that hypometabolism have probably occurred in the slow growing population [28,29]. Similarly, several genes associated with regulation of apoptotic process (BOP1, AIF2L) and proteolysis (NEC, Cathepsin F, ST14, ECE1L, TLL, caspase, TPSB2L, Trypsin, Stromelysin 2, MMPs, Cathepsin F, Coagulation factor XI) were down-regulated in AJ_2S, consistent with the speculation of strong metabolic rate suppression. The suppression of proteolysis pathways were also detected in hypometabolism of other studies, such as aestivating sea cucumber and land snail *Otala lacteal*, and anoxic embryos of the brine shrimp *Artemia franciscana* [30–32]. Depending on species and season, hypometabolism is named differently, such as hibernation, overwintering, torpor, estivation or diapause. Given the global hypometabolism and physiology variation in the slow growing population, such as stopping feeding and activity, intestine degradation and dark skin, we raised the hypothesis that growth retardation in individual growth variation of sea cucumber is one type of dormancy which is used to be against to adverse circumstances. Further investigations will help to clarify the specific mechanisms.

In the present study, peroxidase, an antioxidant enzyme containing four selenium-cofactors that catalyzes the breakdown of hydrogen peroxide and organic hydroperoxides, showed up-regulated expression in AJ_2S. This implied that oxidative stress appeared to be more intense in the slow growing population and peroxidase may participate in antioxidant defenses although further studies are needed to clarify the specific mechanisms. Interestingly, the expression level of most gene transcripts involved in transforming growth factor beta (TGFB) signaling pathway, a multifunctional pathway that controls proliferation, differentiation, and other functions in many cell types, presented reverse trends between AJ_2S vs. AJ_2L and AJ_2S vs. AJ_1S [33,34]. For example, the key ligands or inhibitors in this pathway, such as TGFBI, BMP and follistatin, showed up regulated expression in AJ_2S vs. AJ_2L, while the opposite occurs in AJ_2S vs. AJ_1S. This difference may account for the variation of regulation level of TGFB signaling pathway in different growth stage of sea cucumber, and the mechanisms need to be fully elucidated in future studies.

It is noteworthy that 152 up-regulated unigenes and 241 down-regulated unigenes have low homology to known sequences in public databases, suggesting that they may represent non-coding RNA, misassembled contigs or unknown genes of sea cucumber involved in growth variation [25]. Identification of these above genes and their expression variation provided new understanding about the complex processes of individual growth variation. The information gained from these genes in sea cucumber can be applied to this species to relieve growth variation. Additionally, the differently expressed genes found in this study can be used as potential molecular markers for sea cucumber selective breeding to improve germplasm level.

DEGs were found to be significantly enriched in KEGG metabolic pathways and signal transduction pathways (Table 1). The pathways distribution between the up- and down-regulated genes is quite different. These predicted pathways are likely to be useful in further investigations on their functions. Genes related to “ribosome biogenesis in eukaryotes”, “glycerolipid metabolism”, “fatty acid degradation”, “one carbon pool by folate” and “glutathione metabolism” were concentrated in the up-regulated gene cohort. Genes related to “ECM-receptor interaction”, “focal adhesion”, “PI3K-Akt signaling pathway”, “biosynthesis of amino acids” and many pathways in metabolism category (carbon metabolism, glycerophospholipid metabolism, alpha-Linolenic acid metabolism, linoleic acid metabolism, ether lipid metabolism, arachidonic acid metabolism, fatty acid metabolism) were concentrated in down-regulated gene cohort, which were a lot more than those in the up-regulated gene cohort. Although several genes related to “ribosome biogenesis in eukaryotes” were up-regulated, protein synthesis was not enhanced as the majority of metabolism pathway related genes were down-regulated, which suggested the presence of other specific regulating pathways.

The DEGs and pathways enriched were always found to be relative. The pathways distribution defined by KEGG, consistent with the results of DEG analysis, revealed the clustering of DEGs in cell junction pathway and related pathways, such as “ECM-receptor interaction” and “focal adhesion”, which serve an important role in tissue and organ morphogenesis and in the maintenance of cell and tissue structure and communication. This is consistent with the results from dormancy in human breast cancer which found that STAT3and NFκB expression was induced and whose activation signatures were significantly associated with KEGG pathways related to ECM receptor interactions and focal adhesion [35]. Our results suggest that cell junction and related pathways may play an essential role in the process of individual growth variation.

In comparison of expression levels between AJ_2S vs. AJ_2L and AJ_2S vs. AJ_1S, more down-regulated DEGs were found in AJ_2S vs. AJ_1S than AJ_2S vs. AJ_2L, the majority of which were associated with metabolic process. In the life history of sea cucumber, the nutrient and energy intake were mainly used for metabolism and development in the first 2~3 years. When it comes to aquaculture system, this process is accelerated. Therefore, the global level of gene expression in AJ_1S was enhanced compared to AJ_2L.

The body wall and intestine are two key tissues in the development of sea cucumber, which exhibited different variation in individual growth variation. The body wall often shrinked into a more tight morphology with dark skin. Whereas, the intestine degenerate to just a fraction of their former mass, or disappeared completely. In the present study of AJ_2S, the cell communication related DEGs and pathways were found to be down-regulated in body wall, however, and the metabolic process related DEGs and pathways were universal down-regulated in intestine. These differences could be associated with the physiological change of body wall and intestine in individual growth variation.

## Conclusions

In conclusion, de novo transcriptome sequencing was performed on sea cucumber individuals with growth variation using Illumina sequencing platform. This study identified some important genes (such as cubilin, FGFR1, Hyalin, Fibropellin-1 and Inhibin β) related to immunity defense and hypometabolism, which would benefit researches of variation in individual growth. More importantly, these results highlighted a complex network of metabolic and immunological pathways in the individuals with growth variation. These results will provides deep insight into the molecular basis of individual growth variation in marine invertebrates, and be valuable for understanding some of the physiological differences of process of growth. Our data set provided useful resource for further genetic and genomic studies, and should help support the selective breeding of improved strains of sea cucumber.

## Supporting information

S1 Fig Workflow of library construction and library construction. (PNG)

S2 Fig Length distribution of transcript. (PNG)

S3 Fig Length distribution of assembled unigenes. (PNG)

S1 Table GO classification for unigenes of sea cucumber. (XLSX)

S2 Table KEGG classification for unigenes of sea cucumber. (XLSX)
