# Supplementary figures and images for: Transcriptome analysis of the sea cucumber (Apostichopus japonicus) with variation in individual growth
Source: PLoS One. 2017 Jul 17;12(7):e0181471. doi: 10.1371/journal.pone.0181471 (PMC5513535; doi:10.1371/journal.pone.0181471)

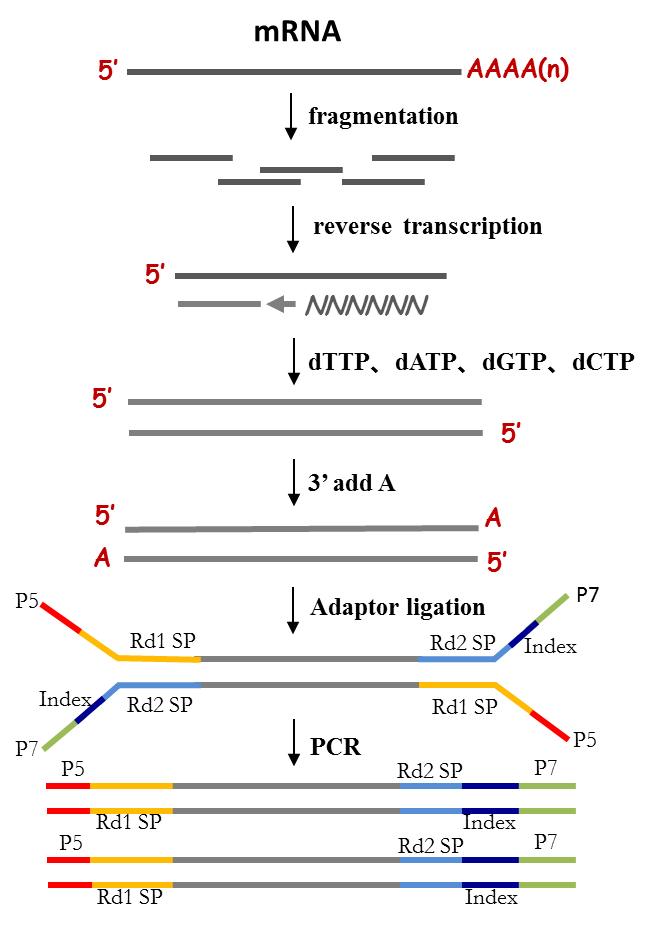

Supplement: S1 Fig — (PNG) [file pone.0181471.s001.png]

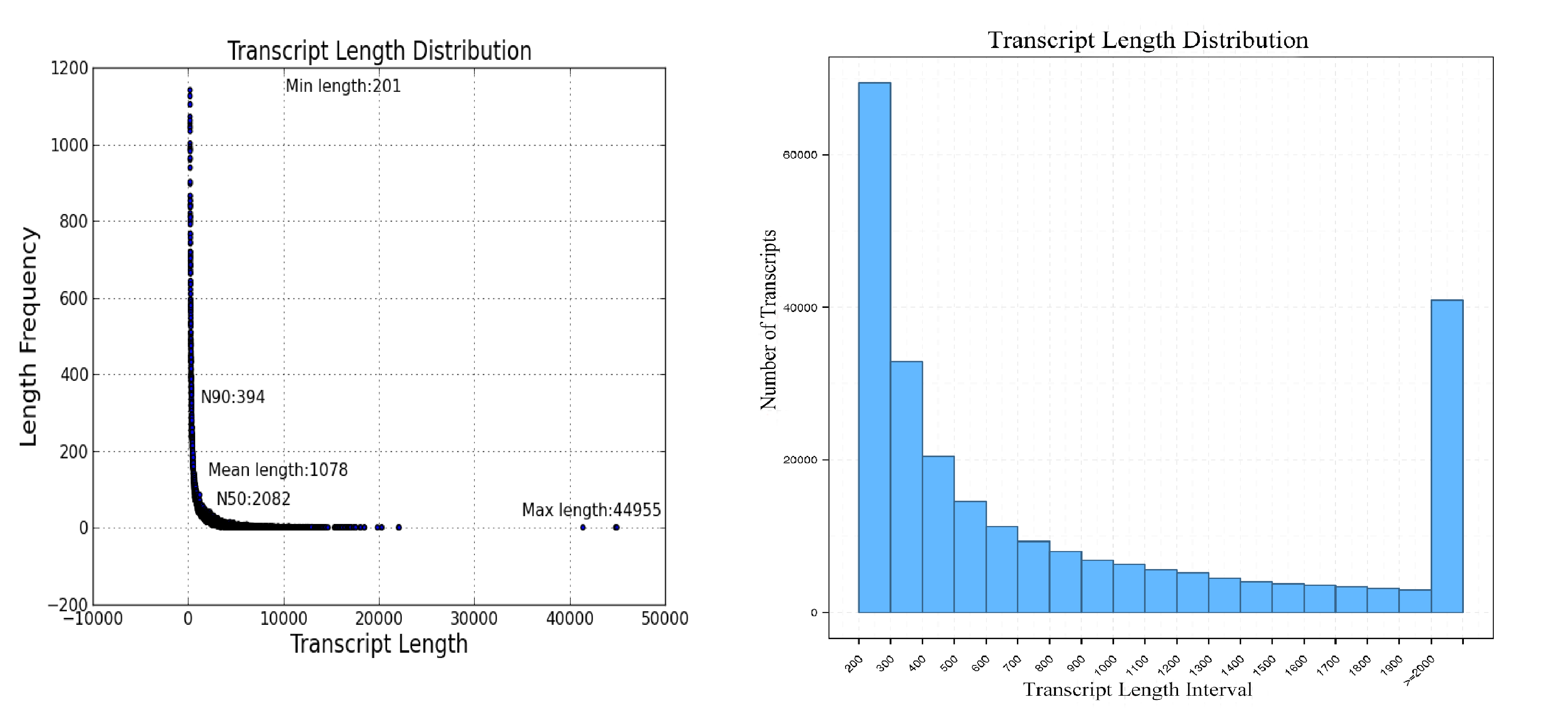

Supplement: S2 Fig — (PNG) [file pone.0181471.s002.png]

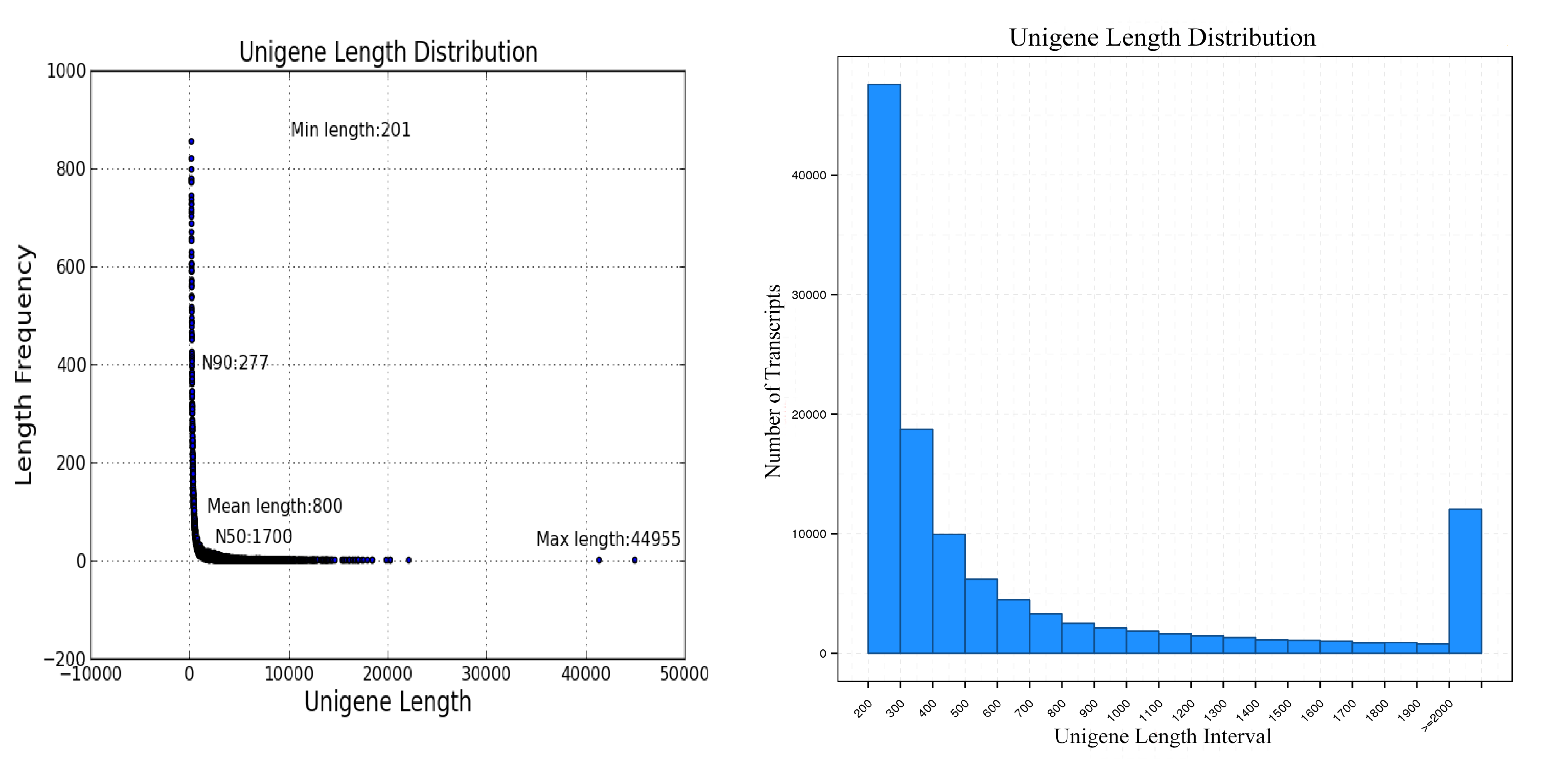

Supplement: S3 Fig — (PNG) [file pone.0181471.s003.png]
